# X-ray nano-tomography of complete scales from the ultra-white beetles Lepidiota stigma and Cyphochilus

**DOI:** 10.1038/s41597-020-0502-y

**Published:** 2020-05-29

**Authors:** Stephanie L. Burg, Adam L. Washington, Julie Villanova, Andrew J. C. Dennison, Daragh McLoughlin, Oleksandr O. Mykhaylyk, Pete Vukusic, Will Furnass, Richard A. L. Jones, Andrew J. Parnell, J. Patrick A. Fairclough

**Affiliations:** 1 Department of Physics and Astronomy, The University of Sheffield, Sheffield, S3 7RH UK; 2 ISIS Pulsed Neutron and Muon Source, Rutherford Appleton Laboratory, Harwell Science and Innovation Campus, Didcot, OX11 0QX UK; 3 ID16B - ESRF - The European Synchrotron, CS 40220, 38043 Grenoble, Cedex 9 France; 4 Akzo-Nobel, Decorative Paints Research and Development, Slough, SL2 5DS UK; 5 Department of Chemistry, Dainton Building, The University of Sheffield, Sheffield, S3 7HF UK; 6 School of Physics, Exeter University, Exeter, EX4 4QL UK; 7 Department of Computer Science, The University of Sheffield, Sheffield, S1 4DP UK; 8 Department of Mechanical Engineering, The University of Sheffield, Sheffield, S3 7HQ UK

**Keywords:** X-ray tomography, Structural materials, Structure of solids and liquids, Entomology

## Abstract

High resolution X-ray nano-tomography experiments are often limited to a few tens of micrometer size volumes due to detector size. It is possible, through the use of multiple overlapping tomography scans, to produce a large area scan which can encompass a sample in its entirety. Mounting and positioning regions to be scanned is highly challenging and normally requires focused ion beam approaches. In this work we have imaged intact beetle scale cells mounted on the tip of a needle using a micromanipulator stage. Here we show X-ray holotomography data for single ultra-white scales from the beetles *Lepidiota stigma* (*L. stigma*) and *Cyphochilus* which exhibit the most effective scattering of white light in the literature. The final thresholded matrices represent a scan area of 25 × 70 × 362.5 µm and 25 × 67.5 × 235µm while maintaining a pixel resolution of 25 nm. This tomographic approach allowed the internal structure of the scales to be captured completely intact and undistorted by the sectioning required for traditional microscopy techniques.

## Abstract (summary)

**Table:** 

Measurement(s)	chitin • scale
Technology Type(s)	computed tomography • x-ray holotomography
Sample Characteristic - Organism	Lepidiota • Cyphochilus

Machine-accessible metadata file describing the reported data: 10.6084/m9.figshare.12251450

## Background & Summary

X-ray tomography is an experimental technique that generates a 3-D reconstruction of the sample from 2D X-ray projection images taken at a multitude of angles ranging from complete rotations to a limited tilt series depending on the beam setup and the sample environment. This powerful technique allows the user to probe the internal structure and morphology of samples that would normally be inaccessible by traditional microscopy techniques without careful sample preparation and sectioning. Nowadays, 3D nano-imaging faces two issues: (i) quite long acquisition time (from minutes to hours using synchrotron sources and hours to days using a lab source) and(ii) limitation of the field of view to several tens of µm, which in some cases is not a representative area or is not sufficient to study the interplay between short and long range properties of the material. Nevertheless, the field of view can be artificially increased by performing successive tomographies along the sample. The development of higher brilliance X-ray synchrotron sources at Max IV, the ESRF and the planned upgrade at Diamond will lead to increases in beam flux and likely decrease the acquisition time per single projection image, which is currently one of the speed limitations for tomography acquisition. Further reductions in acquisition times are being achieved through the use of faster cameras, such as CMOS cameras, which reduce the read out time for each image. Therefore, while still a time-consuming process, large area mapping via x-ray nano-tomography is becoming more attractive and achievable.

In this study, the authors aim to demonstrate that X-ray nano-tomography has the potential to be used for generating cohesive large area 3-D data sets by stitching together overlapping X-ray holotomography scans on full intact single scales from the beetles *Lepidiota stigma* (*L. sitgma*) and *Cyphochilus*. These samples were chosen for their unique optical properties, in that they are highly reflective whilst also ultra-thin as the result of a porous network within the scales composed of chitin and air. These scales have been widely studied via a variety of techniques in the literature^1–5^, including a 2017 study by Wilts *et al*.^6^ in which cryo X-ray nano-tomography which was used to generate a single 343 µm^3^ cube from a column which had been milled from a *Cyphochilus* scale using a focussed ion beam (FIB). However, recent results have shown that the scales crumple and distort when cut open, meaning that traditional microscopy images and analysis performed on sectioned scales are likely unreliable^7^. The results presented in this article are the first one reporting entire and intact scales without external intervention on the internal structure due to sample preparation or degradation during measurements.

The final data sets for the intact scales are matrices which have been thresholded into pixels of either chitin or air. The matrices correspond to a real space volume of ≈4 × 10^5^ µm^3^ for the *Cyphochilus* and ≈6 × 10^5^ µm^3^ for the *L. stigma*. Clear differences between the observed morphology of the scales in the data set generated in this study as compared to previous literature results. For example, the filling fraction or percent of chitin vs air of the internal network was found to be 31 ± 2% for *Cyphochilus* and 34 ± 1% for *L. stigma* in contrast to previous measurements that overestimated the density at ~45–70% for the *Cyphochilus* and ~50% for the L. stigma. This discrepancy is due to the internal network being incredibly fragile and its collapse upon sectioning would result in a densification of the structure. This highlights the importance of examining fragile biological samples intact as well as the advantages of having large area statistics which lend a much greater confidence to calculations such as density.

The data sets generated in this study provides researchers with a detailed example and straightforward methodology to utilize X-ray nano-tomography as more than a small area technique at high resolution and to image large areas and assemble a complete matrix from overlapping tomography scans. Having full representative 3-D volumes will make our understanding of many materials systems much clearer, especially when these data sets are made widely available. Additionally, as the intra-scale structure captured by these extensive whole scale data sets exhibit unique optical properties, they will be of interest to those working on the simulation and design of advanced photonic materials.

## Methods

### X-ray holotomography of single scales

**Fig. 1:**
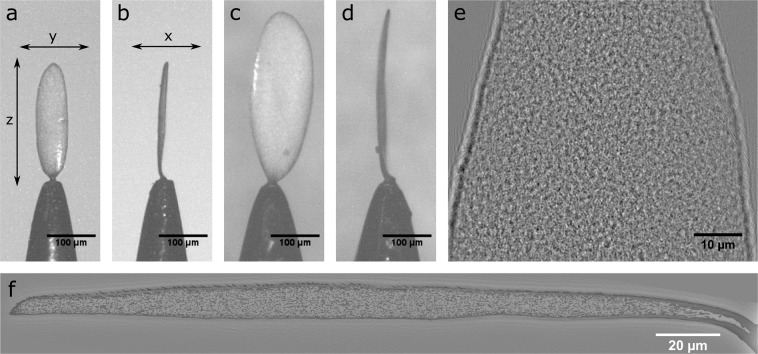
Various optical, x-ray and reconstructed images of the beetle scales. (**a,b**) The mounted Cyphochilus scale *including a* coordinate system *for reference*. (**c,d**) A mounted L. stigma scale. (**e**) An X-ray projection image of scan 2 of the Cyphochilus scale. (**f**) A single slice through the xz plane of the assembled and aligned Cyphochilus matrix.

Performing X-ray nano-holotomography and keeping the highest resolution, requires the sample to be mounted in such a way that it can rotate 360° in the beam completely unobstructed by the sample mount. Therefore, a single scale for *Cyphochilus* and *L. stigma* beetles were mounted on the tip of a needle. This was accomplished using a 3-axis optical alignment stage (Thorlabs) to precisely manipulate a needle, dipped in a UV-curable adhesive (Norton Optical Adhesive), which was cured using UV once the needle was in contact with the beetle scale. The final mounted samples are shown in Fig. 1a–d. While this mounting technique does require a certain amount of practice to become proficient, it does not use any specialised equipment making it accessible to even a casual tomography user. One of its main advantages is a higher throughput in sample preparation, which will prove important given the synchrotron upgrades taking place around the world.

Data for the scales was collected on the beamline ID16B^8^ at the ESRF. The phase contrast image technique used is holotomography which means that 4 tomographies are acquired, each one having a propagation distance slightly different than the first one. The difference in the sample position was 1 mm between the first and second scan, 5 mm between the second and the third and 15 mm between the third and the fourth. For each tomography scan, 3000 projection images with a field of view of 64 µm horizontally and 54 µm vertically were acquired on a PCO Edge 5.5 CMOS camera which has a pixel size of 25 nm. The X-ray incident beam had an energy of 17.5 keV and a cone beam setup was employed over the 360° rotation scan^9^. As the aim of this experiment was to capture 3D data for entire scales, multiple tomography scans, each overlapping each other by 10 µm, were taken starting at the top of each scale and labelled numerically. The *Cyphochilus* scale was ≈240 µm in length and required 6 holotomography scans. As the *Cyphochilus* scale was ≈65 µm wide, the entire scale fit in the field of view of the camera with the exception of a few areas where the very edges were cut off. The *L. stigma* scale was approximately 350 µm in length and therefore required 8 holotomography scans. Unfortunately, the *L. stigma* scale had a maximum width of ≈125 µm meaning it was much larger than the width of the field of view. It was decided to just scan through the centre of the scale from top to bottom, which would exclude the edges on either side, but would only require the assembly of the matrix in the vertical direction maximizing the chances of obtaining a final cohesive matrix.

The raw projection images were processed to the final 3D data set on the ESRF cluster, using standard ESRF instrument routines which included the GNU Octave programming environment (http://www.octave.org), the public domain image analysis program ImageJ (http://rsbweb.nih.gov/ij/) and the Python programming language (https://www.python.org/). These routines included different steps: (i) the alignment of the projection images at each propagation distance, (ii) phase retrieval calculation, (iii) axis alignment, and (iv) final reconstruction of the 3D volume. The alignment of the projection images consists of the resizing and alignment of the individual radiographs recorded at the four distances. A specialised padding scheme, which takes into account the geometry of the sample cross-section as determined from the fourth distance with the largest field of view, is also employed at this stage to reduce the artefacts arising from the edges of the scale which were sometimes temporarily outside the field of view^9^. Unfortunately, due to the scales disordered internal network and overall lack of distinct features (Fig. 1e) the traditional image alignment algorithms provided by the ESRF at that time: real-space cross-correlation and a (FFT)-based cross-correlation, failed to generate reliable results across the four image distances. As a result, the resized projection images for every 100 angles were manually aligned and the pixel displacements were fitted to a polynomial. The manual alignment results were within the ESRF specifications that the fit deviate by no more than 3 pixels for a given angle.

The second step, the phase retrieval calculation, was performed on the aligned images using a contrast transfer function algorithm^10^ and resulted in the calculation of a single phase contrast image per angle. Those images are used to obtain the final 3D volume (2560 × 2560 × 2160 pixels) where each pixel represented a single greyscale value representative of the refractive index of the material, using a filtered back projection algorithm with the ESRF High Speed Tomography in Python (PyHST2) software^11^. The greyscale level of a particular 3D pixel, is directly proportional to the electron density and therefore the mass density in that location^12^. The greyscale values for the reconstruction were stored as 32-bit single precision floats which resulted in a matrix size of ~56 GB per completed tomography.

### Assembly and alignment

Once all the final tomography scans had been reconstructed, the next step was to assemble the individual overlapping scans into a single matrix containing an entire beetle scale. In order to reduce the memory requirements for this step each tomography scan was down-sampled to 8-bit integers which reduced the matrix size of each tomography by a factor of four to ~14 GB. The next step in data processing was to find the overlapping frame between each adjacent scan in order to assemble a single matrix containing the entire beetle scale. Each scan overlapped by 10 µm in z, such that the bottom of one scan and the top of the next, theoretically had 400 frames in common. In order to avoid potential artefacts which could have arisen due to the material outside the field of view at the extreme top and bottom of the scans, the common frames were chosen from the centre of the overlap region. The selection of the overlap frames was accomplished by manually examining slices for a constant z value and locating near identical frames in each scan. Once the corresponding z frames were identified, lateral adjustments were made by eye to ensure as seamless a transition as possible between scans. Complete matrices were then assembled for each of the scales.

Though every care was taken to ensure the samples were mounted as straight as possible, small rotational corrections were still required to align the scale with the x,y and z axes of the matrix. The required rotational angles were determined by examining a single slice through the matrix for each 2D plane. The rotations were carried out using a cubic spline interpolation between pixels. Figure 1f is a single slice through the xz-plane of the completely assembled and rotated *Cyphochilus* matrix.

### Filtering and thresholding

The target dataset for the single scales was a matrix thresholded into pixels of either chitin (dark pixels) or air (light pixels). This involved several image processing steps^13^ as follows.

**Fig. 2:**
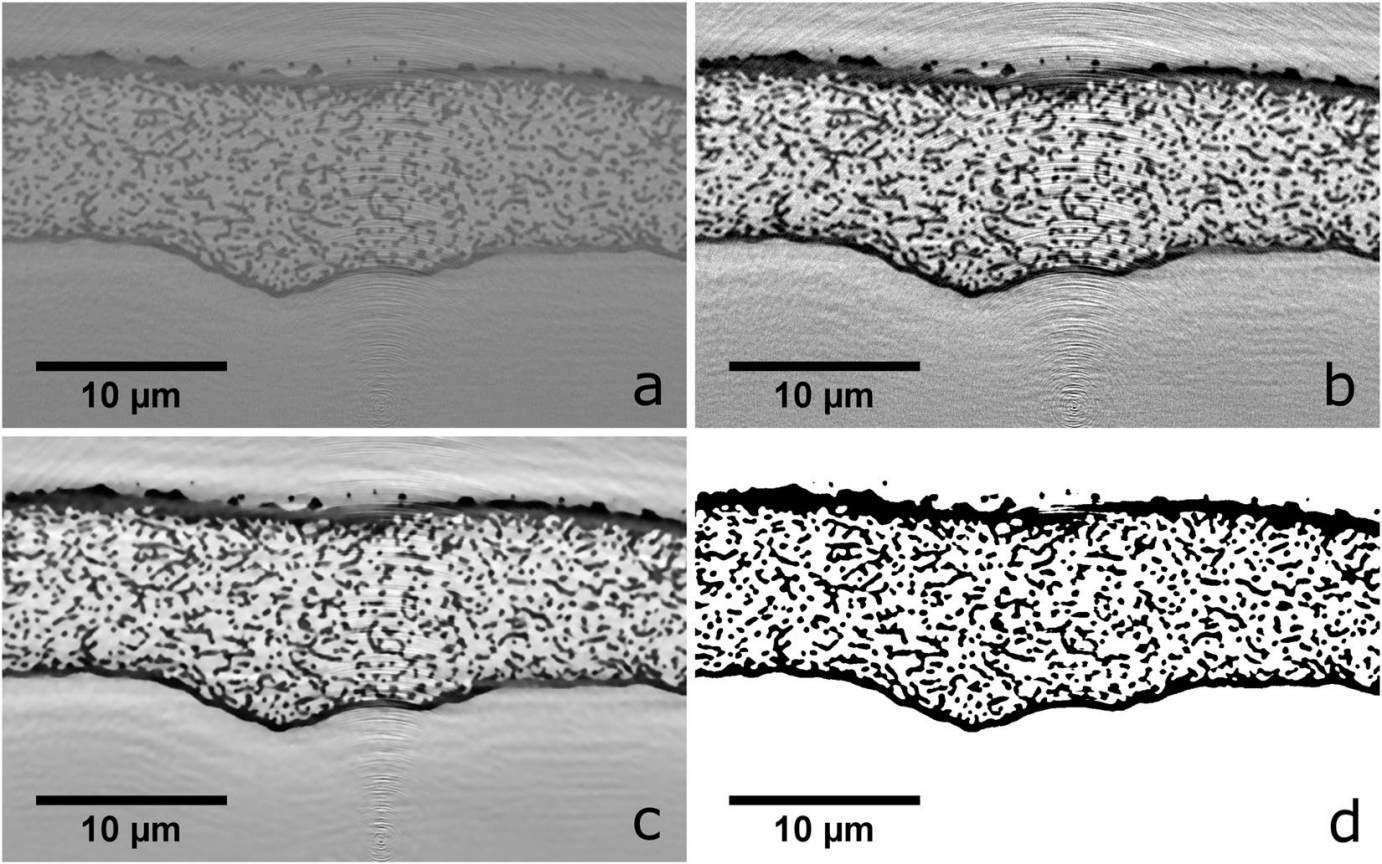
Image analysis steps for achieving a thresholded matrix. A cropped single slice in the xy plane of the *Cyphochilus* matrix that (**a**) has the original greyscale values, (**b**) has had the pixel values saturated, (**c**) has been filtered using the *non-local means denoising* filter, and (**d**) has been thresholded using a combination of single value and adaptive Gaussian thresholding.

First, the matrices were saturated such that the pixel values of the darkest and the lightest 0.5% of the greyscale values were set to the maximum greyscale value for the 99.5 percentile and the minimum greyscale value for the 0.5 percentile as shown in Fig. 2a,b. This was carried out because these extreme greyscale values can most likely be attributed to experimental noise. Second, the matrices were filtered using a *non-local means denoising* filter (Fig. 2c) which effectively preserves sharp edges in the images while successfully reducing the ring artefacts from reconstruction^14^. This algorithm has two main input parameters, the patch size and the patch distance. The patch size, in pixels, tells the algorithm the patch size used for denoising while the patch distance tells the algorithm the maximum distance from the pixel of interest to search for similar pixels^15^. This algorithm is freely available as a part of the scikit-image processing module for Python^16^.

The non-local means denoising filter is computationally intensive; as the matrices to be filtered were ~25 and 40 GB respectively for the *Cyphochilus* and *L. stigma* scales, it was not possible to filter the matrices on a single central processing unit (CPU). Instead, each matrix was divided into overlapping segments which could be run independently. The patch size used for filtering the tomography data was 5 pixels and the patch distance was 7 pixels. Therefore, in order to eliminate edge effects between matrix sections, each section of the matrix needed to overlap with its adjacent sections by more than 7 pixels. An overlap of 10 pixels on all sides was used and the *Cyphochilus* and *L. stigma* matrices were divided into 1,504 sections and 2,320 sections each of which required ~40 minutes to run and ~1.5 GB of memory and were able to be processed in parallel on the Sheffield Advanced Research Computer (ShARC) in less than a week.

The final step in the image processing routine was to threshold the matrix. To capture the internal structure of the scales an adaptive Gaussian thresholding routine was used where the weighted mean of the surrounding pixels was computed based on a user defined region size by applying a Gaussian filter (available as a part of the scipy.ndimage package for Python^17^). The thresholding value for each pixel was then computed as the weighted mean minus an offset. The relevant parameters used were a region size of 60 pixels, an offset intensity of 0.06 and the standard deviation for the Gaussian kernel, σ, was computed according to Eq. 1.

1 $$\sigma =(region\,size-1)/6$$

**Fig. 3:**
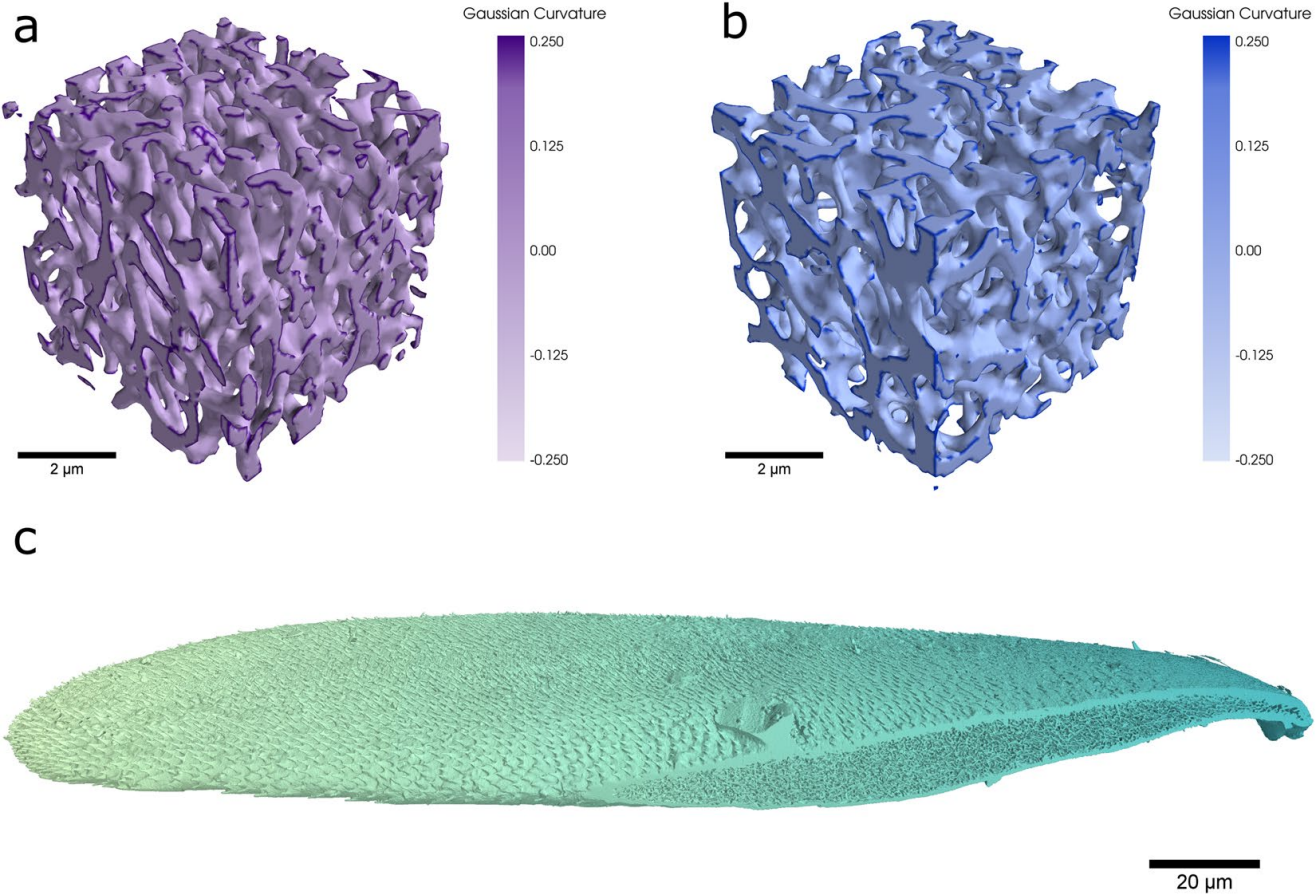
Three dimensional renders of the tomography data. (**a**) A render of a 5 µm cube of the internal structure of the *Cyphochilus* scale which has been shaded according to the computed Gaussian curvature for the mesh vertices. (**b**) A 5 µm cube of the internal *L. stigma* scales also coloured by Gaussian curvature. (**c**) A 3-D render of the final complete matrix for the *Cyphochilus* scale. At the bottom right of the scale, where the internal nano-structure is visible, is the result of a small section at the edge of the scale being outside the field of view of the tomography scan when the data was collected. The scale has been artificially coloured for aesthetic purposes.

While the adaptive Gaussian thresholding was effective at picking out the internal structure, as it is sensitive to sharp changes in greyscale values, it was unable to pick out areas of solid chitin. To overcome this, a simple single value thresholding was applied where only pixels with a normalized greyscale intensity of less than 0.4 were considered to be chitin. This meant that the single value thresholding only encompassed the darkest pixels in the matrix. The final thresholding shown in Fig. 2d is the result of multiplying the Gaussian thresholding matrix by the matrix generated by a single value thresholding. This allowed both the internal structure and the edge cuticle of the scale to be faithfully represented in the final thresholded matrix. While the images in Fig. 2 are from the Cyphochilus scale matrix, the exact same thresholding was used for the *L. stigma* matrix. Additional images of the thresholded matrices for both scales are given in Supplementary Figs. 1 and 2. Images showcasing 3-D renders of the thresholded matrices for the internal structures of both scales and the entire Cyphochilus scale are shown in Fig. 3. For an animated .gif of Fig. 3c please see Supplementary Movie 1.

## Data Records

The data sets referenced in this paper have been deposited at Figshare^18^. The following matrices are provided for both the *Cyphochilus* (CY) and *L. stigma* (LS) scales. The original consecutive overlapping tomography scans, post reconstruction on the ESRF cluster, are listed in numerical order (CY_1-6 and LS_1-8). The complete scale matrices with the original greyscale values after being assembled and aligned (CY_greyscale and LS_greyscale). The complete thresholded scale matricies post saturation, filtering and thresholding (CY_thresholded and LS_thresholded). Two unique slabs of the internal scale cut from the complete matrix (CY_slab_1-2 and LS_slab-1-2) and a representative 5 µm cube of the internal structure (CY_cube and LS_cube) which had the least mean squared error compared to the average directional correlation functions of the 275 cubes sampled. All matrices have a real space to pixel ratio of 25 nm/pixel and in the case of the thresholded matrices, 0 is scale material and 1 is air. The sizes listed for each matrix are for an unpacked and/or unzipped matrix.

## Technical Validation

Prior to the acquisition of the data on ID16B the alignment procedures of the beamline were carefully carried out by the local beamline contact who was also responsible for optimising the experimental conditions and advising on phase calculation and reconstruction procedures. While the alignment of the projection images was done by eye, and therefore could be considered subjective, the quality of the reconstruction was vastly improved when manual alignment was used, compared to the results from the automated algorithms, as demonstrated in Supplementary Fig. 3. Therefore, the authors are confident the tomography data processed using manual alignment represents the best final quality reconstructions based on the original data.

One of the major concerns when performing X-ray tomography at high energies is sample degradation. This was offset by using small exposure times per projection angle (0.15 s). There is strong evidence that there was no significant sample degradation, as it proved possible to locate the same frame in each overlapping scan. If the sample was appreciably degrading in the beam, by the time the area was rescanned a second time, observable differences would have been seen in a frame to frame comparison (Supplementary Fig. 4). This was evidently not the case, as it proved possible to assemble the individual data sets to create a near seamless final matrix.

All of the image processing steps used to achieve the final thresholded matrices were carefully chosen and optimised by the best judgment of the authors. However, the original reconstructed tomography scans have been included in the data repository should anyone wish to process and threshold the data differently.

## Usage Notes

The data sets which are <1.5 GB, which include the slabs and cubes cut from the full scale matrices, have been simply left as .npy files and can be loaded into Python using numpy.load(). For the larger data sets, all of the greyscale data has been zipped before uploading into a Python .npz format. To load the greyscale data simply load the .npz file using numpy.load() which will load the data into a dictionary like object with a single element, the greyscale matrix, which can be called with the default key of ‘arr_0’. An example script to load greyscale matrices has been provided in the README.txt. The thresholded data sets have been packed into uint8 arrays prior to zipping. Therefore, once loaded from the .npz file, the thresholded matrix must be unpacked using numpy.unpackbits() and then reshaped into a 3D matrix according to the appropriate values in Tables 1 and 2. An example script for loading and unpacking the thresholded data is included in the README.txt.

**Table 1:** Details of the tomography data sets provided for the Cyphochilus (CY) scale.

File name	Size (pixels)	Size (um)	Size (GB)	Image type
CY_1	2560 × 2560 × 2160	64 × 64 × 54	14.2	Greyscale (0-254)
CY_2	2560 × 2560 × 2160	64 × 64 × 54	14.2	Greyscale (0-254)
CY_3	2560 × 2560 × 2160	64 × 64 × 54	14.2	Greyscale (0-254)
CY_4	2560 × 2560 × 2160	64 × 64 × 54	14.2	Greyscale (0-254)
CY_5	2560 × 2560 × 2160	64 × 64 × 54	14.2	Greyscale (0-254)
CY_6	2560 × 2560 × 2160	64 × 64 × 54	14.2	Greyscale (0-254)
CY_greyscale	1000 × 2700 × 9400	25 × 67.5 × 235	25.4	Greyscale (0-254)
CY_thresholded	1000 × 2700 × 9400	25 × 67.5 × 235	25.4	Thresholded (0-1)
CY_slab_1	200 × 800 × 1000	5 × 20 × 25	1.28	Thresholded (0-1)
CY_slab_2	200 × 800 × 1000	5 × 20 × 25	1.28	Thresholded (0-1)
CY_cube	200 × 200 × 200	5 × 5 × 5	0.064	Thresholded (0-1)

**Table 2:** Details of the tomography data sets provided for the L. stigma (LS) scale.

File name	Size (pixels)	Size (um)	Size (GB)	Image type
LS_1	2560 × 2560 × 2160	64 × 64 × 54	14.2	Greyscale (0-254)
LS_2	2560 × 2560 × 2160	64 × 64 × 54	14.2	Greyscale (0-254)
LS_3	2560 × 2560 × 2160	64 × 64 × 54	14.2	Greyscale (0-254)
LS_4	2560 × 2560 × 2160	64 × 64 × 54	14.2	Greyscale (0-254)
LS_5	2560 × 2560 × 2160	64 × 64 × 54	14.2	Greyscale (0-254)
LS_6	2560 × 2560 × 2160	64 × 64 × 54	14.2	Greyscale (0-254)
LS_7	2560 × 2560 × 2160	64 × 64 × 54	14.2	Greyscale (0-254)
LS_8	2560 × 2560 × 2160	64 × 64 × 54	14.2	Greyscale (0-254)
LS_greyscale	1000 × 2800 × 14500	25 × 70 × 362.5	40.6	Greyscale (0-254)
LS_thresholded	1000 × 2800 × 14500	25 × 70 × 362.5	40.6	Thresholded (0-1)
LS_slab_1	200 × 800 × 1000	5 × 20 × 25	1.28	Thresholded (0-1)
LS_slab_2	200 × 800 × 1000	5 × 20 × 25	1.28	Thresholded (0-1)
LS_cube	200 × 200 × 200	5 × 5 × 5	0.064	Thresholded (0-1)

## Supplementary information

Supplementary Information

Supplementary Movie 1

## Data Availability

The ESRF High Speed Tomography in Python (PyHST2) software which was used to reconstruct the phase images is open source and can be found at: https://software.pan-data.eu/software/74/pyhst2. The current pipeline for processing the raw data prior to its use in the PyHST2 algorithm is a large collection of scripts in MATLAB, Python and GNU Octave which makes it difficult to bundle into a single tomography pipeline. However, the ESRF is currently working to convert all of the scripts to Python to create a completely open source pipeline, though additional computing power, such a high performance computing cluster will likely be necessary. All additional image processing was done using open source Python libraries; these have been noted at the appropriate stages in the text.
