# Supplementary material for: X-ray nano-tomography of complete scales from the ultra-white beetles Lepidiota stigma and Cyphochilus
Source: Sci Data. 2020 May 29;7:163. doi: 10.1038/s41597-020-0502-y (PMC7260169; doi:10.1038/s41597-020-0502-y)
Supplement: Supplementary file 1 — Supplementary Information [file 41597_2020_502_MOESM1_ESM.pdf]

Table of Contents

Supplementary Figure 1 1
Supplementary Figure 2 2
Supplementary Figure 3 3
Supplementary Figure 4 3

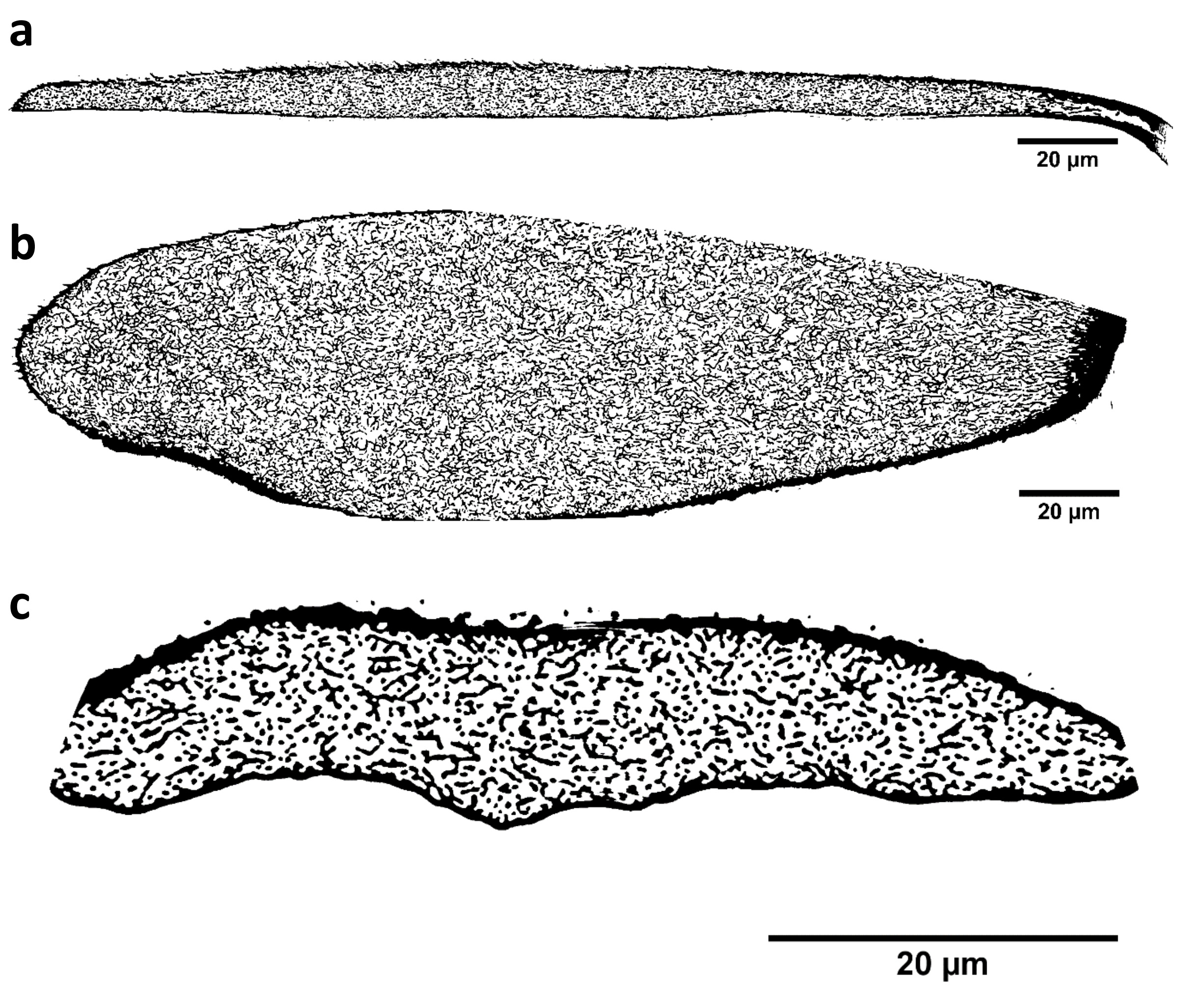

Figure 1: Slices through various planes of the thresholded matric for the *Cyphochilus* scale. (a) The xz plane, (b) the yz plane, and (c) the xy plane.

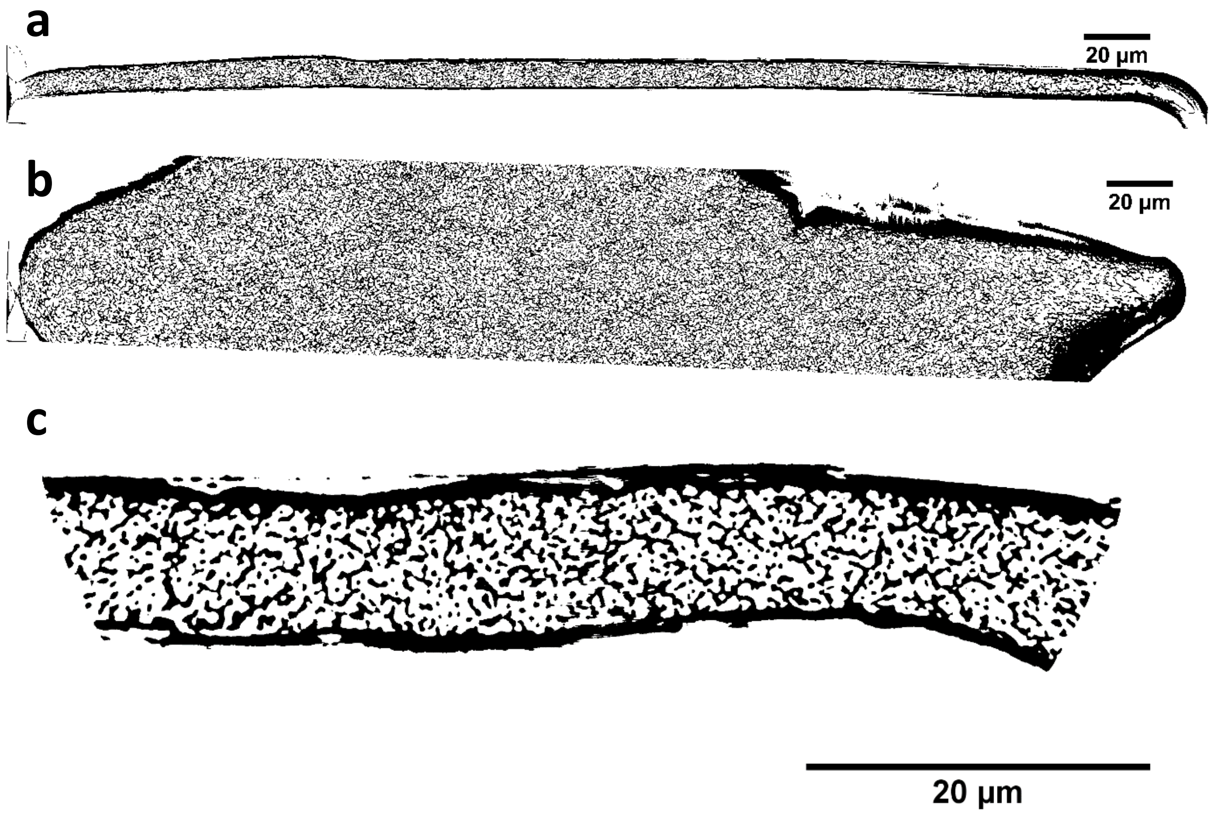

Figure 2: Slices through various planes of the thresholded matric for the *L. stigma* scale. (a) The xz plane, (b) the yz plane, and (c) the xy plane.

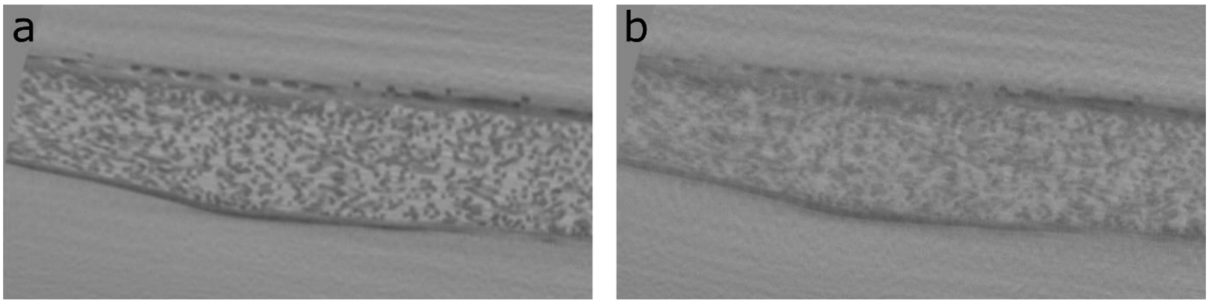

Figure 3: A comparison of manual versus automated image alignment methods. (a) A single slice through the *Cyphochilus* reconstruction processed using manual image alignment. (b) The same slice but processed instead using an automated (FFT)-based cross-correlation.

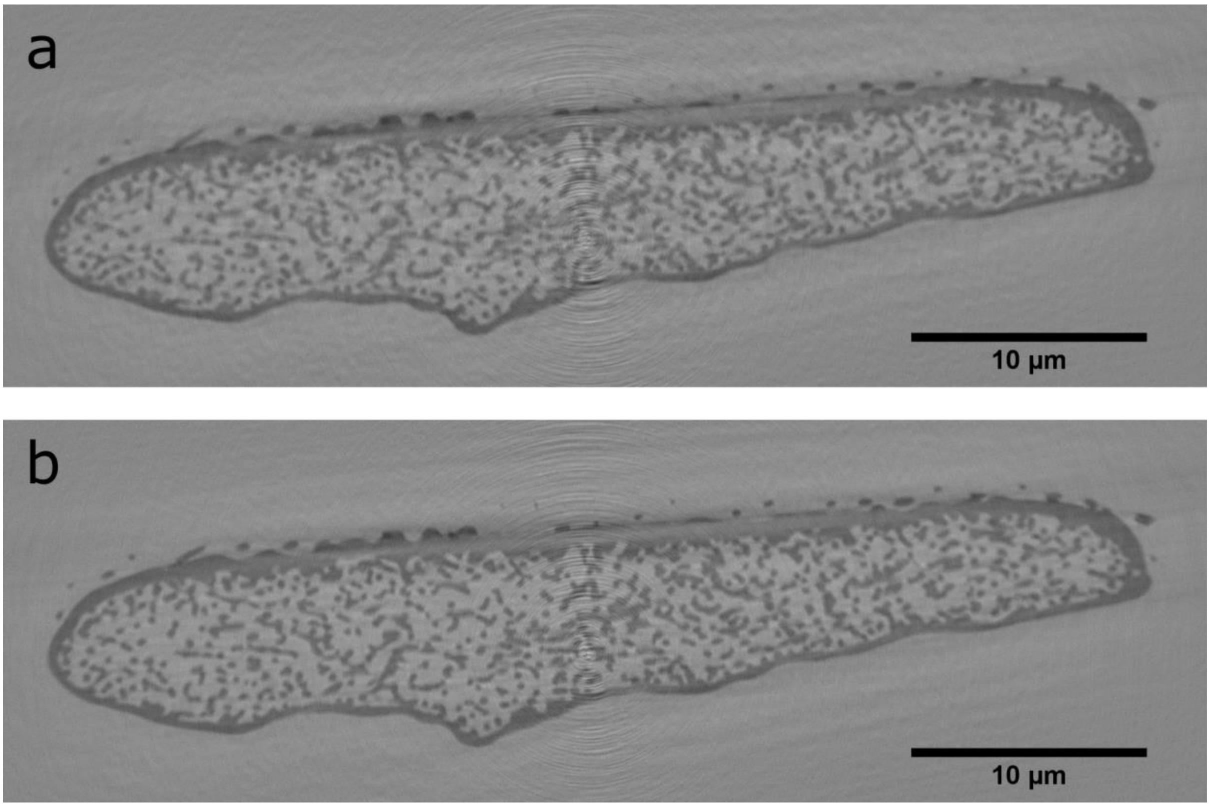

Figure 4: The same frame in two different tomography scans taken on the same area of the scale. (a) CY_A frame 2010 in z (b) CY_B frame 256 in z.
