# Supplementary figures and images for: X-ray nano-tomography of complete scales from the ultra-white beetles Lepidiota stigma and Cyphochilus
Source: Sci Data. 2020 May 29;7:163. doi: 10.1038/s41597-020-0502-y (PMC7260169; doi:10.1038/s41597-020-0502-y)

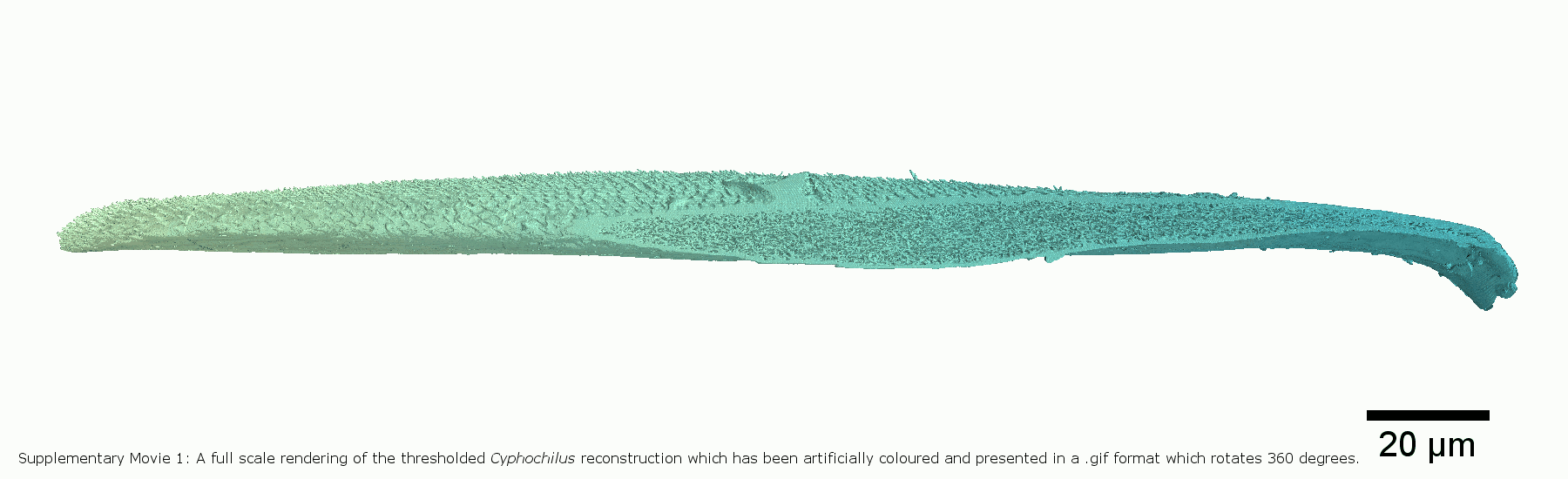

Supplement: Supplementary file 2 — Supplementary Movie 1 [file 41597_2020_502_MOESM2_ESM.gif]
